# Increased ERCC1 expression is linked to chromosomal aberrations and adverse tumor biology in prostate cancer

**DOI:** 10.1186/s12885-017-3489-9

**Published:** 2017-07-26

**Authors:** Frank Jacobsen, Billurvan Taskin, Nathaniel Melling, Charlotte Sauer, Corinna Wittmer, Claudia Hube-Magg, Martina Kluth, Ronald Simon, Dirk Pehrke, Burkhard Beyer, Thomas Steuber, Imke Thederan, Guido Sauter, Thorsten Schlomm, Waldemar Wilczak, Katharina Möller, Sören A. Weidemann, Susanne Burdak-Rothkamm

**Affiliations:** 1 Institute of Pathology, University Medical Center Hamburg-Eppendorf, Martinistr. 52, 20246 Hamburg, Germany; 2 General, Visceral and Thoracic Surgery Department and Clinic, University Medical Center Hamburg-Eppendorf, Hamburg, Germany; 3 Martini-Clinic, Prostate Cancer Center, University Medical Center Hamburg-Eppendorf, Hamburg, Germany; 4 Department of Urology, Section for translational Prostate Cancer Research, University Medical Center Hamburg-Eppendorf, Hamburg, Germany

**Keywords:** ERCC1, DNA repair, Prostate cancer, Prognosis

## Abstract

**Background:**

Animal model experiments have suggested a role of the DNA repair protein ERCC1 (Excision Repair Cross-Complementation Group 1) in prostate cancer progression.

**Methods:**

To better understand the impact of ERCC1 protein expression in human prostate cancer, a preexisting tissue microarray (TMA) containing more than 12,000 prostate cancer specimens was analyzed by immunohistochemistry and data were compared with tumor phenotype, PSA recurrence and several of the most common genomic alterations (*TMPRSS2:ERG* fusions: deletions of *PTEN*, 6q, 5q, 3p).

**Results:**

ERCC1 staining was seen in 64.7% of 10,436 interpretable tissues and was considered weak in 37.1%, moderate in 22.6% and strong in 5% of tumors. High-level ERCC1 staining was linked to advanced pT stage, high Gleason grade, positive lymph nodes, high pre-operative serum PSA, and positive surgical margin status (*p* < 0.0001 each). High ERCC1 expression was strongly associated with an elevated risk of PSA recurrence (*p* < 0.0001). This was independent of established prognostic features. A subgroup analysis of cancers defined by comparable quantitative Gleason grades revealed that the prognostic impact was mostly driven by low-grade tumors with a Gleason 3 + 3 or 3 + 4 (Gleason 4: ≤5%). High ERCC1 expression was strongly associated with the presence of genomic alterations and expression levels increased with the number of deletions present in the tumor. These latter data suggest a functional relationship of ERCC1 expression with genomic instability.

**Conclusion:**

The results of our study demonstrate that expression of ERCC1 - a potential surrogate for genomic instability - is an independent prognostic marker in prostate cancer with particular importance in low-grade tumors.

**Electronic supplementary material:**

The online version of this article (doi:10.1186/s12885-017-3489-9) contains supplementary material, which is available to authorized users.

## Background

Prostate cancer is the most common cancer in males in the western societies. While most patients will never suffer symptoms from their disease, prostate cancer is still the third most common cause of cancer related death of men in most Western countries [1]. The highly variable clinical course of the disease cannot be predicted reliably enough by currently available criteria such as Gleason grade, clinical stage and PSA value. Additional and better prognostic markers are needed to differentiate between aggressive high risk and non-aggressive low risk cancer subtypes in order to prevent unnecessary invasive treatments.

The DNA repair endonuclease ERCC1 (Excision Repair Cross-Complementation Group 1) catalyzes 5′ incision during nucleotide excision repair process (NER) [2, 3]. ERCC1 has been described to be physiologically expressed in several tissues including skin, breast, intestine, testis, and ovary [4]. Overexpression of ERCC1 has been found in many cancer types such as urothelial carcinoma [5], head and neck squamous cell carcinoma [6] and non-small cell lung cancer [7]. For these entities it has been proposed that ERCC1 overexpression may serve as a prognostic and/or predictive tumor marker [5–9].

ERCC1 is of potential interest in prostate cancer. Experimental data from a mouse model system suggested an altered ERCC1 function as potential driver for an invasive prostate cancer phenotype [10]. Moreover, a specific nucleotide polymorphism of the ERCC1 gene was linked to prostate cancer aggressiveness in a Spanish cohort study of 494 men [11]. The present study evaluates the clinical impact of ERCC1 expression in human prostate cancer. For this purpose, a preexisting prostate cancer tissue microarray was examined for ERCC1 expression by immunohistochemistry.

## Methods

### Patients

Twelve thousand four hundred twenty seven prostatectomy specimens were obtained from consecutive patients treated between 1992 and 2012 in the Department of Urology and the Martini Clinics at the University Medical Center Hamburg-Eppendorf. Tumor stage, Gleason grade, nodal stage and the resection margin status were recorded. Classical Gleason categories and “quantitative” Gleason grading was performed as described [12]. Follow-up data were available for a total of 12,344 patients (median 36 months, range 1 to 241 months; Table 1). Prostate specific antigen (PSA) recurrence was defined as a postoperative PSA of ≥0.2 ng / ml and increasing. All prostate specimens were embedded for histological analysis by a standard procedure [13]. The TMA was produced as described [14, 15]. In brief, one 0.6 mm core sample was taken from a representative tissue block and distributed among 27 TMA blocks, each with 144 to 522 samples. Each TMA block contained various control and normal prostate tissue. The molecular database attached to this TMA contained results on ERG expression, *ERG* break apart FISH analysis [16], deletion status of 5q21 (*CHD1*) [17], 6q15 (*MAP3K7*) [18], *PTEN* (10q23) [19–21] and 3p13 (*FOXP1*) [22]).

**Table 1:** Pathological and clinical data of the arrayed prostate cancers

	No. of patients (%)	
	Study cohort on TMA (*n* = 12,427)	Biochemical relapse among categories
Follow-up (mo)		
n	11,665 (93.9%)	2769 (23.7%)
Mean	62.9	−
Median	50.0	−
Age (y)		
≤50	334 (2.7%)	81 (24.3%)
51–59	3061 (24.8%)	705 (23%)
60–69	7188 (58.2%)	1610 (22.4%)
≥70	1761 (14.3%)	370 (21%)
Pretreatment PSA (ng/ml)		
<4	1585 (12.9%)	242 (15.3%)
4–10	7480 (60.9%)	1355 (18.1%)
10–20	2412 (19.6%)	737 (30.6%)
>20	812 (6.6%)	397 (48.9%)
pT stage (AJCC 2002)		
pT2	8187 (66.2%)	1095 (13.4%)
pT3a	2660 (21.5%)	817 (30.7%)
pT3b	1465 (11.8%)	796 (54.3%)
pT4	63 (0.5%)	51 (81%)
Gleason grade		
≤3 + 3	2848 (22.9%)	234 (8.2%)
3 + 4	6679 (53.8%)	1240 (18.6%)
3 + 4 Tert.5	433 (3.5%)	115 (26.6%)
4 + 3	1210 (9.7%)	576 (47.6%)
4 + 3 Tert.5	646 (5.2%)	317 (49.1%)
≥4 + 4	596 (4.8%)	348 (58.4%)
pN stage		
pN0	6970 (91%)	1636 (23.5%)
pN+	693 (9%)	393 (56.7%)
Surgical margin		
Negative	9990 (81.9%)	1848 (18.5%)
Positive	2211 (18.1%)	853 (38.6%)

Percent in the column “Study cohort on TMA” refers to the fraction of samples across each category. Percent in column “Biochemical relapse among categories” refers to the fraction of samples with biochemical relapse within each parameter in the different categories. NOTE: Numbers do not always add up to 12,427 in different categories because of cases with missing data. Abbreviation: AJCC, American Joint Committee on Cancer

### Immunohistochemistry

Newly cut sections of the complete TMA were stained on the same day in a single experiment. Slides were deparaffinized and antigen was retrieved by heat (5 min at 121 °C, pH 7.8 Tris-EDTA-citrate buffer). ERCC1 specific mouse monoclonal antibody clone UMAB8, BioCAT GmbH, Heidelberg; cat#UM500008; dilution 1:150) was applied at 37 °C for 60 min. Bound antibody was visualized with the EnVision Kit (Dako, Glostrup, Denmark). ERCC1 typically stained 100% tumor cell nuclei in a single tissue spot. Staining intensity was assessed semi-quantitatively as negative, weak, moderate and strong.

### Statistics

Contingency tables were calculated to analyze associations between ERCC1 expression and clinico-pathological parameters. Chi-square (Likelihood) test was employed to identify significant relationships between these parameters. The F-test was used in analysis of variance to detect differences of the mean of groups. Kaplan-Meier curves were generated for the event of PSA recurrence free survival and the log-Rank test was applied to test for significant differences between stratified survival curves. The prognostic significance of pathological, molecular and clinical parameters was assessed by Cox proportional hazards regression analysis. All calculations were done with JMP® software (SAS Institute Inc., NC, USA).

## Results

### Technical issues

A total of 11,665 (93.9%) patients had follow up data and 10,436 (84%) of samples were interpretable in the TMA analysis (Table 1). Reasons for non-informative cases were lack of tissue samples (1991 spots; 16%), absence of unequivocal cancer tissue in the TMA spot or missing data.

### ERCC1 immunohistochemistry

**Fig. 1:**
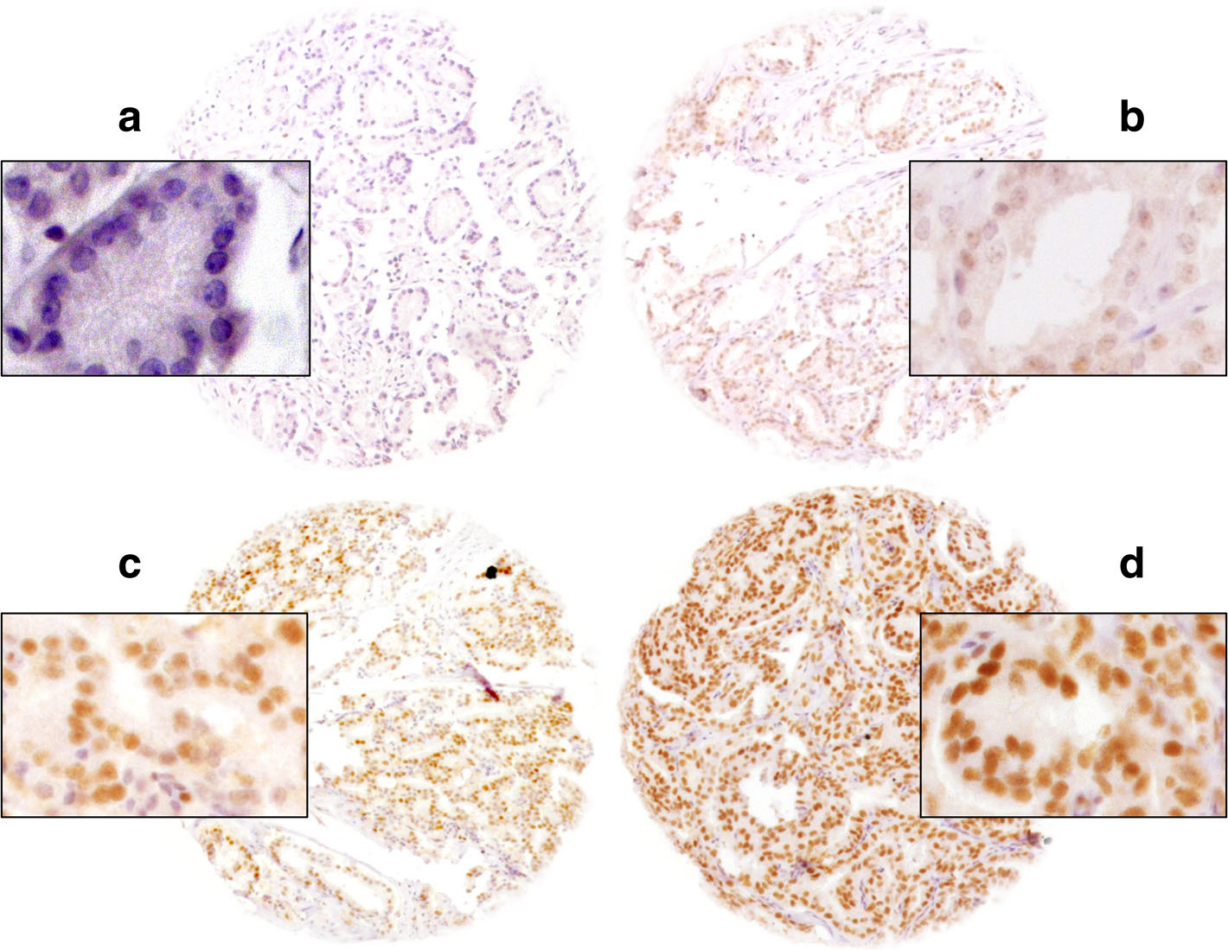
Representative pictures of **a**) negative, **b**) weak, **c**) moderate and **d**) strong ERCC1 staining in prostate cancer

ERCC1 staining was negative or weak in basal and luminal cells of normal prostate glands. Positive nuclear ERCC1 staining was seen in 64.7% of 10,436 interpretable tissue samples, and was graded as weak in 37.1%, moderate in 22.6%, and strong in 5% of tumors. Representative images of ERCC1 immunohistochemistry in prostate cancer samples are shown in Fig. 1. Strong ERCC1 staining was linked to advanced pT stage, high Gleason grade, positive lymph nodes, high pre-operative serum PSA measurement, and positive surgical margin status (*p* ≤ 0.0078; Table 2).

**Table 2:** Association between ERCC1 staining results and prostate cancer clinical characteristics

	ERCC1 (%)					
Parameter	n evaluable	Negative	Weak	Moderate	Strong	*p value*
All cancers	10,436	35.4	37.1	22.6	5.0	
Tumor stage						
pT2	6790	38.4	37.3	20.1	4.2	<0.0001
pT3a	2299	31.6	35.5	26.4	6.6	
pT3b-pT4	1308	26.4	38.8	28.5	6.3	
Gleason grade						
≤3 + 3	2363	46.3	34.2	16.5	3.0	<0.0001
3 + 4	5630	34.7	37.4	22.9	5.0	
3 + 4 Tert.5	368	33.4	40.8	22.0	3.8	
4 + 3	1040	25.6	38.8	28.4	7.3	
4 + 3 Tert.5	563	23.4	40.0	29.1	7.5	
≥4 + 4	466	25.8	38.2	29.0	7.1	
Lymph node metastasis						
N0	5856	32.7	37.6	23.9	5.8	0.0037
N+	585	25.6	39.8	27.9	6.7	
Preoperative PSA level (ng/ml)						
<4	1293	32.6	40.5	21.9	4.9	0.0078
4–10	6256	35.2	37.8	22.1	4.8	
10–20	2058	37.5	33.9	23.5	5.1	
>20	714	35.6	33.9	24.8	5.7	
Surgical margin						
Negative	8294	36.1	37.5	21.8	4.7	<0.0001
Positive	1953	32.7	35.5	25.9	5.9	

### Association with TMPRSS2:ERG fusion status

**Fig. 2:**
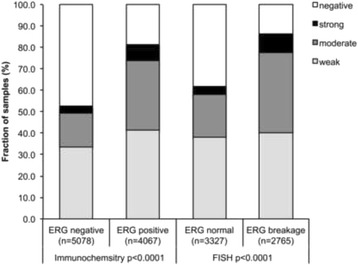
Positive ERCC1 staining correlates with ERG staining in immunochemistry or ERG breakage in fluorescence in situ hybridization (FISH)

ERCC1 expression was massively linked to the presence of ERG expression and rearrangement. At least weak ERCC1 staining was found in 85.4% of cancers with immunohistochemically detected ERG expression and in 81.4% of tumors with *ERG*-rearrangement, but only in 52.6% (IHC) or 61.8% (FISH) of ERG-negative cancers (*p* < 0.0001 each, Fig. 2). ERCC1 staining was similarly linked to unfavorable tumor phenotype in subsets of both ERG-negative and ERG-positive cancers (Additional file 1: Tables S1 and S2).

### Associations with key genomic changes of prostate cancer

**Fig. 3:**
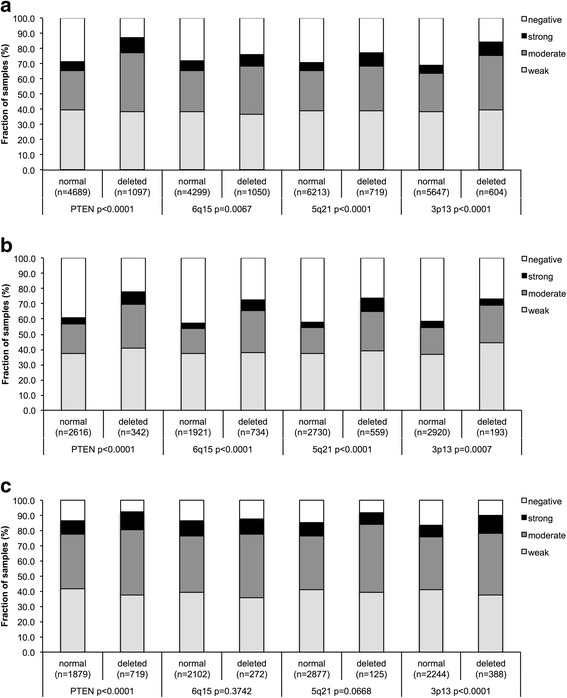
Association between positive ERCC1 staining and 10q23 (PTEN), 5q21 (CHD1), 6q15 (MAP3K*7*), and 3p13 (*FOXP1*) deletion in **a**) all cancers, **b**) the ERG-negative and **c**) ERG-positive subset

**Fig. 4:**
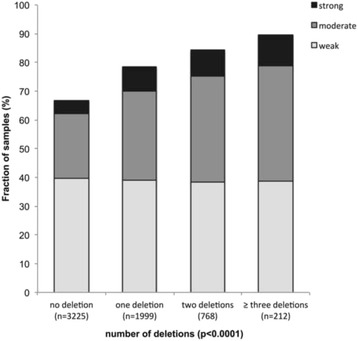
Association between positive ERCC1 staining and number of deletions in the ERG-positive subset

Chromosomal deletions represent the most frequent genomic changes in prostate cancer next to *TMPRSS2:ERG* fusion. To study whether ERCC1 expression might be particularly linked to any of the most common deletions, ERCC1 data were compared to preexisting findings on 10q23 (*PTEN*), 3p13 (*FOXP1*), 6q15 (*MAP3K7*) and 5q21 (*CHD1*) deletions (Fig. 3). These analyses showed that ERCC1 expression was strongly linked to all examined deletions. This was particularly evident for ERG negative carcinomas (Fig. 3b) and only marginally visible in ERG positive carcinomas (Fig. 3c). Moreover, the level of ERCC1 expression was also related to the number of deletions found in all cancer (deletion load, Fig. 4; *p* < 0.0001). This held true also in the subset of ERG-negative and ERG-positive cancers (*p* < 0.0001; data not shown).

### Association with cell proliferation

High levels of ERCC1 staining were significantly linked to increased tumor cell proliferation measured as Ki67 labeling index (Ki67LI) (Table 3, *p* < 0.0001). This association held also true in almost all subgroups of cancers with identical Gleason grade (≤3 + 3; 3 + 4; 4 + 3; *p* < 0.0001 each).

**Table 3:** Association between ERCC1 expression and Ki67-labeling index in all, low- grade and high-grade prostate cancers

Gleason grade	ERCC1	n	Ki67 LI (mean ± SD)	*p value*
All grades	Negative	2189	1.94 ± 0.06	<0.0001
	Weak	2210	2.95 ± 0.06	
	Moderate	1422	3.38 ± 0.07	
	Strong	319	4.02 ± 0.14	
≤3 + 3	Negative	672	1.68 ± 0.08	<0.0001
	Weak	471	2.46 ± 0.09	
	Moderate	233	3.04 ± 0.13	
	Strong	44	2.77 ± 0.31	
3 + 4	Negative	1200	1.89 ± 0.07	<0.0001
	Weak	1308	2.86 ± 0.06	
	Moderate	866	3.27 ± 0.08	
	Strong	190	3.70 ± 0.17	
4 + 3	Negative	241	2.51 ± 0.22	<0.0001
	Weak	342	3.57 ± 0.18	
	Moderate	240	3.87 ± 0.22	
	Strong	65	4.98 ± 0.42	
≥4 + 4	Negative	62	3.79 ± 0.56	0.05
	Weak	80	4.8 ± 0.49	
	Moderate	74	4.28 ± 0.51	
	Strong	19	6.89 ± 1.01	

### Associations with prostate-specific antigen recurrence

**Fig. 5:**
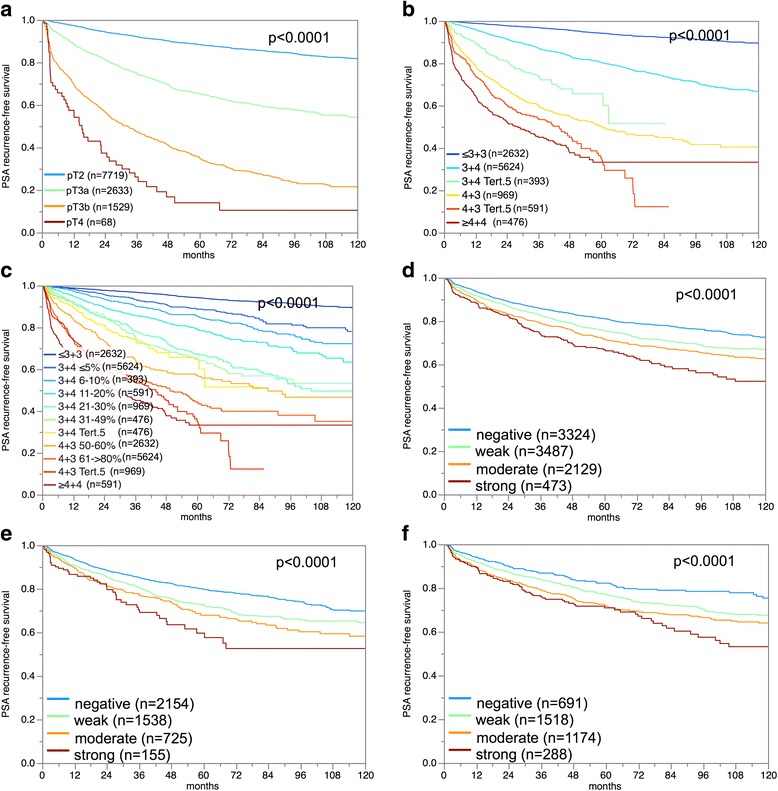
Prostate specific antigen (PSA) recurrence free survival correlates with **a**) pathological stage, **b**) classical Gleason grade, **c**) quantitative Gleason grade, and ERCC1 expression in **d**) all cancers, **e**) the ERG-fusion negative and **f**) positive subset

**Fig. 6:**
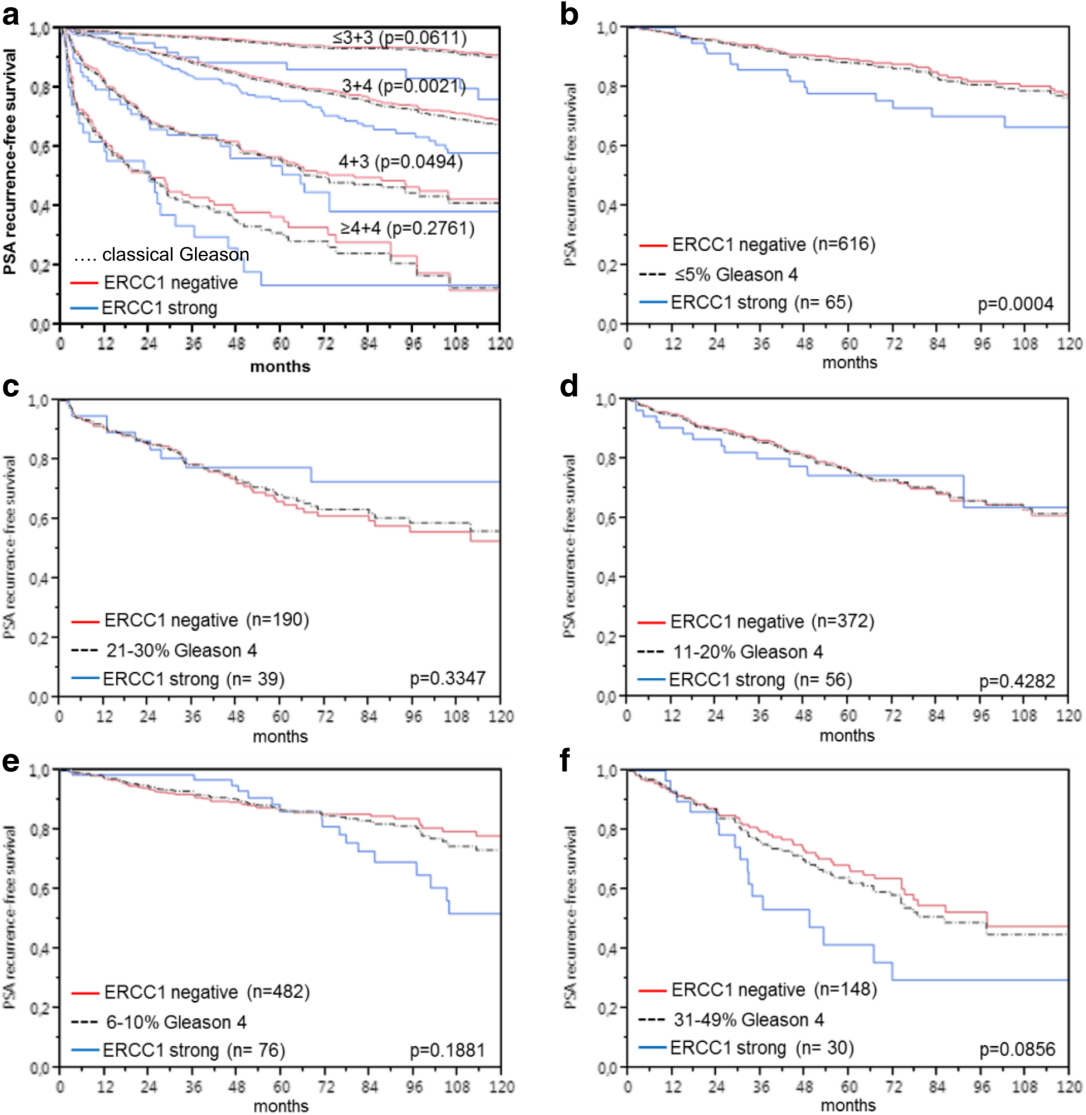
Limited prognostic impact of ERCC1 expression in cancers grouped by **a**) classical and **b-f**) quantitative Gleason score. The quantitative Gleason score is defined by the percentage of Gleason 4 patterns. Black dotted line denotes Gleason score category result, red line negative and blue line strongly positive ERCC1 cancers within the respective category

The prognostic impact of pT stage (Fig. 5a), traditional Gleason grade (Fig. 5b), and quantitative Gleason grade (Fig. 5c) were strongly linked to PSA recurrence. There was a significant association between high ERCC1 staining levels and early PSA recurrence (*p*< 0.0001; Fig. 5d). This held also true for the subgroups of ERG-negative (*p*< 0.0001; Fig. 5e) and ERG-positive (*p*< 0.0001; Fig. 5f) cancers. Analyzing subsets of tumors with comparable traditional and quantitative Gleason grades revealed that ERCC1 expression measurement did not provide very much additional prognostic impact in morphologically characterized tumor sets. Significant associations with PSA recurrence were seen in Gleason 3 + 3 = 6 (*p* = 0.061), Gleason 3 + 4 (*p* = 0.0021) and 4 + 3 carcinomas (*p* = 0.0494) but not in tumors with a Gleason ≥4 + 4 (Fig. 6a). A further refined subgroup analysis by quantitative Gleason grading showed that high ERCC1 expression identified cancers with worse outcome only in those 3 + 4 carcinomas with a minimal fraction of Gleason 4 (≤5%) (Fig. 6b; *p* = 0.0004). None of the other groups with a comparable quantitative Gleason grade showed outcome differences according to the ERCC1 status (Fig. 6c-f).

### Multivariate analysis

Four different scenarios were tested. All these analyses were also done in the ERG-negative and ERG-positive subset (Table 4). Scenario 1 used the post-operatively available parameters (pathological tumor stage (pT), lymph node status (pN), surgical margin status, pre-operative PSA value and classical Gleason grade). In Scenario 2 the nodal status was dropped to reduce missing data as lymph node dissection is not yet standardized in radical prostatectomy. Scenario 3 included ERCC1 expression, pre-operative PSA, clinical tumor stage (cT stage) and Gleason grade obtained on the prostatectomy specimen. Since post-operative determination of a tumors Gleason grade is more precise than the preoperatively determined Gleason grade [23], scenario 4 was added to better model the pre-operative situation. Here, the pre-operative biopsy Gleason grade was combined with pre-operative PSA, cT stage and ERCC1 expression. Overall, these scenarios suggest a relevant role of ERCC1 expression as a prognostic factor, which tended to be - especially in the pre-operative setting - independent of established factors (scenario 3 and 4).

**Table 4:** Multivariate analysis including ERCC1 expression in all cancers, ERG-negative and ERG-positive subset

Tumor subset	Scenario	n analyzable	*p value*							
			Preoperative PSA-Level	pT Stage	cT Stage	Gleason grade prostatectomy	Gleason grade biopsy	pN Stage	R Stage	ERCC1-Expression
All cancers	1	5644	0.0009	<0.0001	-	<0.0001	-	<0.0001	0.0008	0.0746
	2	9193	<0.0001	<0.0001	-	<0.0001	-	-	<0.0001	0.0052
	3	9062	<0.0001	-	<0.0001	<0.0001	-	-	-	0.0045
	4	8926	<0.0001	-	<0.0001	-	<0.0001	-	-	<0.0001
ERG-negative subset	1	2829	<0.0001	<0.0001	-	<0.0001	-	0.0001	0.0846	0.1110
	2	4471	<0.0001	<0.0001	-	<0.0001	-	-	0.0002	0.0184
	3	4432	<0.0001	-	<0.0001	<0.0001	-	-	-	0.0526
	4	4368	<0.0001	-	<0.0001	-	<0.0001	-	-	<0.0001
ERG-positive subset	1	2242	0.0057	<0.0001	-	<0.0001	-	0.0763	0.0092	0.0626
	2	3584	<0.0001	<0.0001	-	<0.0001	-	-	<0.0001	0.0613
	3	3506	<0.0001	-	<0.0001	<0.0001	-	-	-	0.0746
	4	3451	<0.0001	-	<0.0001	-	<0.0001	-	-	0.0009

## Discussion

In this study increased expression of the DNA repair factor ERCC1 was identified as a strong prognostic marker in prostate cancer, in particular for low-grade tumors. Under the selected experimental conditions, detectable ERCC1 staining was found in 65% of prostate tumors. ERCC1 expression was virtually not detected in normal prostate epithelium. This finding suggests an up-regulation of ERCC1 during tumor development in a proportion of prostate cancers. So far, comprehensive studies on ERCC1 expression in clinical prostate cancer samples are lacking. However, high-level ERCC1 expression has been reported from the prostate cancer cell lines DU-145 and LNCaP [24]. Also, the 12 prostate cancer samples, included in the Human Protein Atlas, showed ERCC1 staining in 83–100% of cases depending on the antibody used [25].

The strong association of elevated ERCC1 expression with adverse morphological and clinical features of prostate cancer found in this study, argues for a role of ERCC1 overexpression/activation in prostate cancer progression. This assumption is supported by findings in other cancer types where associations between high ERCC1 expression levels and reduced overall survival had been found. This, for example, includes reports on NSCLC as well as in gastric and pancreatic cancers [7, 9, 26].

The large number of samples in this TMA and the associated database with numerous molecular features allowed us to draw conclusions on the mechanistic role of ERCC1 in prostate cancer. ERCC1-mediated endonucleolytic incision and homologous recombination (HR) have been implicated in the repair of DNA-interstrand crosslinks (ICLs) which induce a potent replication block followed by formation and repair of double strand breaks (DSBs) [27]. Defective DSB repair and faulty DNA replication are thought to be involved in the generation of chromosomal aberrations commonly seen in cancer cells [28]. The striking association found between elevated ERCC1 expression and chromosomal deletions as well as with a positive ERG status is suggestive of a link between ERCC1 activation and presence of chromosomal damage. ERCC1 may thus represent a surrogate for genomic instability in proliferative active prostate cancer cells. This hypothesis is further supported by the continuous increase of ERCC1 levels with the number of deletions detected, suggesting high level activity of replication associated DNA damage repair mechanisms in subsets of prostate cancer with generation of chromosomal aberration via DSB formation and faulty repair. *TMPRSS2:ERG* fusions were most strikingly linked to ERCC1 expression. The reason for this particular strong association remains unclear. Earlier studies had not implicated ERCC1 as a gene that is directly regulated by the transcription factor ERG [29–31]. The association between deletions and ERCC1 expression was less clear in ERG positive than in ERG-negative cancers, which is likely due to the (already) markedly elevated levels of ERCC1 in ERG-positive tumors. In case of additional deletions, this may not allow for a further elevation measurable under the experimental conditions applied in this study. The observed strong association between high levels of ERCC1 and rapid tumor cell proliferation, as determined by the Ki67 labeling index, is consistent with the involvement of ERCC1 in the repair of replication associated DNA damage [32] as rapidly proliferating cancer cells are subjected to high replication stress [33, 34].

ERCC1 was an independent predictor of poor outcome in most multivariate analyses suggesting a strong clinical utility of ERCC1 measurement. Remarkably, the analysis of the prognostic role of ERCC1 expression in subgroups of prostate cancer that were narrowly defined by identical quantitative Gleason grades suggested a limitation of the prognostic value of ERCC1 measurement to the earliest lesions, i.e. Gleason 3 + 3 or 3 + 4 with only minimal (≤5%) Gleason 4 fraction. This limitation of the prognostic impact to these subgroups is not a disappointment as these tumors are subject to the most difficult therapeutic decision making with options ranging from active surveillance to prostatectomy. That ERCC1 expression failed to provide prognostic information in most subgroups in cancers with comparable quantitative Gleason findings also demonstrates how high the bar lies for prognostic molecular tests in prostate cancer. The Gleason grading system is purely based on the simple distinction of architectural features, neglects any cytological criteria, but is extremely powerful. The prognostic power of the Gleason grade is much higher than the histologic grading in various other cancer types, such as for example kidney cancer [35] or invasive bladder cancer [36]. This holds true if the Gleason grading method is limited 5 prognostic subgroups [37]. Based on the analysis of a cohort of more than 10,000 prostate cancers available at our institution, we had recently shown, that using the percentage of Gleason 4 grades as a continuous variable could expand Gleason Grade information. Both in biopsies and in prostatectomy samples, prostate cancer prognosis deteriorates gradually with increasing percentage of Gleason 4 pattern (quantitative Gleason Grade) [12]. Given the high impact of pure morphologic information in prostate cancer, we believe that a further improvement of morphologic assessment going beyond architecture and also involving digital image analysis and deep machine learning will play a very important role in prostate cancer assessment in the future.

## Conclusions

In summary, elevated expression of ERCC1 is strongly linked to unfavorable tumor phenotype and PSA recurrence in prostate cancer. In this study, an association between ERCC1 overexpression and chromosomal aberrations (including both ERG fusion and deletions) was observed. These findings suggest overexpression of ERCC1 in the context of replication associated DNA damage repair, genomic instability and generation of structural chromosomal alterations.

## Additional file

Additional file 1: Table S1. Association between ERCC1 immunostaining results and prostate cancer phenotype in ERG-negative tumors. **Table S2.** Association between ERCC1 immunostaining results and prostate cancer phenotype in ERG-positive tumors. (PDF 112 kb)

## Abbreviations

CHD1: Chromodomain-helicase-DNA-binding protein 1
ERCC1: Excision repair cross-complementation
ERG: Erythroblast transformation-specific (ETS) related gene
FISH: Fluorescence in situ hybridization
FOXP1: Forkhead box protein P1
Ki67LI Ki67: Labeling index
MAP3K7: Mitogen-activated protein kinase kinase kinase 7
NER: Nucleotide excision repair
PSA: Prostate specific antigen
PTEN: Phosphatase and tensin homolog
TMA: Tissue microarray
TMPRSS2: Transmembrane protease, serine 2
