# Supplementary material for: Increased ERCC1 expression is linked to chromosomal aberrations and adverse tumor biology in prostate cancer
Source: BMC Cancer. 2017 Jul 26;17:504. doi: 10.1186/s12885-017-3489-9 (PMC5530529; doi:10.1186/s12885-017-3489-9)
Supplement: Additional file 1: Table S1. — Association between ERCC1 immunostaining results and prostate cancer phenotype in ERG-negative tumors. Table S2. Association between ERCC1 immunostaining results and prostate cancer phenotype in ERG-positive tumors. (PDF 112 kb) [file 12885_2017_3489_MOESM1_ESM.pdf]

Table S1 Association between ERCC1 immunostaining results and prostate cancer phenotype in ERG-negative tumors

		ERCC1				
**Parameter**	**n evaluable**	**negative (%)**	**weak (%)**	**moderate (%)**	**strong (%)**	**p value**
**All cancers**	5,078	47.4	33.6	15.7	3.3	
**Tumor stage**						
pT2	3,432	49.2	33.4	14.5	2.9	<0.0001
pT3a	1,007	47.0	32.3	17.2	3.6	
pT3b-pT4	624	38.0	37.0	19.7	5.3	
**Gleason grade**						
≤3+3	1,050	61.0	27.1	10.4	1.5	
3+4	2,695	48.0	34.4	14.9	2.8	
3+4 Tert.5	224	44.6	34.4	17.9	3.1	<0.0001
4+3	533	36.0	36.8	21.4	5.8	
4+3 Tert.5	301	30.2	40.2	21.9	7.6	
≥4+4	271	32.8	36.9	24.4	5.9	
**Lymph node metastasis**						
N0	2,944	44.5	35.2	16.5	3.7	0.0025
N+	275	35.3	36.0	21.8	6.9	
**Preop. PSA level (ng/ml)**						
<4	533	44.8	35.3	15.4	4.5	
4-10	2,998	47.3	34.5	15.3	2.9	0.1597
10-20	1,105	48.5	31.8	16.6	3.2	
>20	399	47.9	30.8	16.0	5.3	
**Surgical margin**						
negative	4,042	47.6	33.9	15.4	3.0	0.0853
positive	948	45.8	32.5	17.4	4.3	

Table S2 Association between ERCC1 immunostaining results and prostate cancer phenotype in ERG-positive tumors

		ERCC1				
**Parameter**	**n evaluable**	**negative (%)**	**weak (%)**	**moderate (%)**	**strong (%)**	**p value**
**All cancers**	4,067	18.6	41.4	32.2	7.8	
**Tumor stage**						
pT2	2,427	20.9	42.9	29.3	6.8	<0.0001
pT3a	1,072	16.6	38.2	35.2	10.1	
pT3b-pT4	552	11.8	41.3	39.1	7.8	
**Gleason grade**						
≤3+3	867	26.9	41.9	25.6	5.7	
3+4	2,307	17.8	41.5	32.6	8.1	
3+4 Tert.5	120	11.7	51.7	30.8	5.8	<0.0001
4+3	399	12.3	39.6	38.1	10.0	
4+3 Tert.5	225	12.4	40.9	38.7	8.0	
≥4+4	147	12.9	36.1	40.1	10.9	
**Lymph node metastasis**						0.3736
N0	2,311	16.1	40.8	34.0	9.1	
N+	251	13.1	44.2	35.5	7.2	
**Preop. PSA level (ng/ml)**						
<4	571	20.1	44.5	28.9	6.5	0.0170
4-10	2,472	18.8	42.1	31.3	7.8	
10-20	730	18.4	37.0	36.2	8.5	
>20	245	13.5	40.4	38.0	8.2	
**Surgical margin**						
negative	3,184	19.5	41.9	30.9	7.6	0.0030
positive	808	15.5	39.5	36.9	8.2	
